# The Effect of Self-care Education Based on Self-efficacy Theory, Individual Empowerment Model, and Their Integration on Quality of Life among Menopausal Women

**DOI:** 10.30476/IJCBNM.2021.86814.1370

**Published:** 2022-01

**Authors:** Mahboobeh Kafaei-Atrian, Zohreh Sadat, Saeideh Nasiri, Fatemeh Sadat Izadi-Avanji

**Affiliations:** 1 Department of Midwifery, School of Nursing and Midwifery, Kashan University of Medical Sciences, Kashan, Iran; 2 Trauma Nursing Research Centre, Kashan University of Medical Sciences, Kashan, Iran; 3 Department of Medical Surgical Nursing, School of Nursing and Midwifery, Kashan University of Medical Sciences, Kashan, Iran

**Keywords:** Empowerment, Menopause, Quality of life, Self-efficacy

## Abstract

**Background:**

Menopause has adverse physical and emotional effects on the quality of life. The aim of this study was to determine the effect of self-care education based on self-efficacy theory, individual empowerment model, and their integration on the quality of life among menopausal women.

**Methods:**

In this quasi-experimental study, 186 menopausal women, aged 45-60 years at Kashan health centers entered the study using cluster sampling for health centers and simple random sampling inside each center from the list of the population from December 2019 to March 2020. The intervention was performed in 3 groups, using empowerment training, self-efficacy training, and their integration for 4 sessions each lasting 1-1.5 hours of weekly training. Three study groups were selected from different centers to minimize information exchange. Menopausal women’s quality-of-life questionnaire was used to assess the quality of life. Questionnaires were completed pre-intervention and one and three months after completing the intervention. SPSS16 software and ANOVA, Chi-square, and repeated measure tests were used.

**Results:**

The mean±SD score of quality of life in pre-intervention measurement was 32.96±10.62 in empowerment, 31.93±12.54 in self-efficacy, and 34.07±11.7 in integrated groups. The intervention was effective in improving the quality of life of all three groups (P values were<0.001 for time, 0.92 for group, and 0.38 for time*group interaction).

**Conclusion:**

This study showed that empowerment and enhancing self-efficacy could improve menopausal women’s quality of life. This can help health professionals to better educate postmenopausal women about self-care in menopausal complications.

## INTRODUCTION

Quality of life according to the World Health Organization (WHO) is people’s understanding of their life situation in terms of culture, the value system in which they live and their goals, expectations, standards and priorities. ^1^ Women spend more than one-third of their life after menopause. ^2^ As a physiological process, menopause is associated with both physical and emotional discomfort and has negative effects on the quality of life. ^3 - 6^ Although menopause is a physiological phenomenon, due to the multiplicity of symptoms and complications, it needs proper cultural education, proper adaptation to problems, and promotion of mental health. ^7^ Intervention to care for postmenopausal women improves their quality of life in terms of vasomotor, sleep and memory problems. ^8^ It was previously shown that menopausal women’s group training strengthens their knowledge, helps them share their experiences and overall understanding of life, and reduces menopausal symptoms. ^9^ In another study, the quality of life score of postmenopausal women improved one month after the educational intervention of life skills. ^10^

Another factor affecting the quality of life is empowerment. Empowering is the social process of recognizing, promoting, and increasing people’s capacity to meet their needs, solve their own problems, and mobilize the resources needed to control their own lives. ^11^ According to Sarah Langwe’s theory, there are five stages in empowerment; they include equity, access, awareness, participation and control. ^12^ Menopausal women need to be empowered to make decisions about their health. ^13 , 14^ It was previously shown that the empowerment intervention improves the quality of life of postmenopausal women, while this change is not observed in the control group. ^15^ Another factor that affects the quality of life is self-efficacy. ^16 , 17^ According to Bandura’s definition, self-efficacy is an individual’s belief in the ability to perform the desired function, and there is a significant direct relationship between quality of life and self-efficacy. ^16 , 18^

Training of postmenopausal women increases their self-efficacy for self-care. ^19 , 20^ Self-care refers to the activities that people do to stay healthy and fight disease. ^21^ Using self-care, people can perform their life activities by their own to survive and maintain and promote their own life. ^22^ Postmenopausal women do not practice enough self-care, and menopausal care training improves their self-care and QOL. ^23 - 25^ However, the teaching method is an important principle that helps to achieve a better result. ^22^ Finally, in previous studies the relationship between empowerment and self-efficacy with self-care and quality of life of postmenopausal women has been investigated separately, but these two factors have not been compared. ^15 , 20^ Moreover, Sarah Lange’s empowerment theory is not used for menopausal self-care. Also, the review study will pave the way for future research on promoting and empowering menopause women’s health. ^13^

Given that women spend much of their lives in postmenopausal years, study on integration of self-efficacy and empowerment may find a way to double self-care in postmenopausal stage and improve their quality of life. Also, the use of Sarah Lang’s empowerment theory may show different results, and helps examine the maintenance effect of self-care educational intervention on the quality of life of postmenopausal women. The aim of this study was to determine the effect of self-care education based on self-efficacy theory, individual empowerment model, and their integration on the quality of life among postmenopausal women.

## MATERIALS AND METHODS

This is a quasi-experimental study on three interventional groups. It was conducted in Kashan health centers from December 2019 to March 2020. The inclusion criteria were Iranian healthy menopausal women according to their report, age between 45-60 years, 1 to 5 years being passed after the last menstrual period, lack of other physiological or pathological reasons for amenorrhea, no use of hormonal drugs or food or plant hormones, and personal consent to participate in the study. Exclusion criteria were absences from the intervention sessions and unwillingness to participate in the study. 

Sample size was determined using the following formula: $N={\left(Z_{1-\alpha /2}+Z_{1-\beta }\right)}^{2}(\sigma_{1}^{2}+\sigma_{2}^{2})/d^{2}$ . According to mean±SD in the vasomotor dimensions of the questionnaire in two study groups (5.4±3.2 in the experimental and 7.6±2.6 in the control groups) in a previous study, α=0.05 and β=0.1, the sample size was determined 37 in each group. ^15^ Because of the existence of three groups in the study and using the formula: nꞋ=√K*n and (k=3-1), the number of samples reached 52. Considering the attrition rate of 20%, the sample size reached 62 women in each group. 

In order to collect the data, Kashan city was firstly divided into three geographical clusters; then, three centers with larger covered population were selected with purposive sampling from each cluster (a total of 9 centers). Centers in each cluster were randomly assigned to self-efficacy, empowerment, or integration groups with simple random method. Thus, each cluster included all the three groups. Each center is dedicated to one intervention group to minimize transferring information between groups. In each center, the samples were selected from the list of covered population using simple random sampling, (with random number table) with similar sample size. Then, the researchers introduced themselves to the participants over the phone and explained the purpose of the research to them. If these people met the entry criteria, they were invited to participate in the study. If they did not meet the inclusion criteria, other women were replaced using the list of covered population randomly.

The first author of the article had a doctorate in health education and health promotion, was in charge of the training plan, and compiled educational materials from reference books and articles available on reputable scientific sites. The educational materials were approved by experts in health education and health promotion. For all three groups, there were two similar and two specific training sessions. In the first two sessions, the principles of menopausal self-care were taught similarly to all three groups. In the second two sessions, special training was presented for each group: 1) in the first group based on empowerment, 2) in the second group based on self-efficacy, and 3) in the third group based on the integration of empowerment and self-efficacy.

The content of the two similar sessions on self-care training included nutrition, exercise, sleep problems, hot flashes, osteoporosis, sexual dysfunction, urinary incontinence, vaginal dryness, changes in appearance, thinning hair, obesity, and fat accumulation in the chest and abdomen. 

As to empowerment training in first group, according to Sarah Langwe’s theory, five stages in empowerment are considered, including equity, access, awareness, participation, and control. ^12^ (Table 1)

**Table 1:** Contents of trainings according to steps of theory or model for each group

Groups	Training	Theoretical steps and model factors
Empowerment	Participants were provided with information about menopausal symptoms and ways to deal with the symptoms, including nutrition, exercise, sleep problems, hot flashes, osteoporosis, sexual dysfunction, urinary incontinence, vaginal dryness, changes in appearance, thinning hair, obesity, and fat accumulation in the chest and abdomen.	Awareness
	The situations in which postmenopausal women can participate for social or professional communication were introduced to them such as sports or religious or educational meetings and planned trips.	Awareness, access, equity
	They learned to use the power of all family members and share responsibilities between family members.	Participation and control
	Freelance centers such as carpet weaving or handicraft centers were introduced.	Access, participation, control
	Health counseling centers, screening, nutrition and psychology and centers providing free medical and dental services were introduced.	Equity, access
	Learned how to prepare foods suitable for their age at a reasonable price (rich in calcium, low in salt, low in fat and low in sugar).	Access, participation, control
	They learned how to receive insurance and free medical services for those who were not insured.	Access
Self-efficacy	Successful participants in self-care were introduced to others.	Mastery experience
	People’s ability to perform a behavior was enhanced by verbal encouragement and emotional/physiological arousal, i.e. the participants were encouraged if they mentioned their self-care experience during the sessions.	Verbal persuasion and somatic and emotional status
	Several short and motivational clips on self-care methods were shown to influence the experiences of succession, verbal persuasion, and emotional/ physiological arousal of learners.	Vicarious experience
	Wherever participants had an opinion, they were free to present it, and other participants were encouraged to participate in the discussion as much as possible.	Vicarious experience
	Whenever participants mentioned self-care activities such as diet or weight loss or exercise, they were asked to express their feelings.	Verbal persuasion and somatic and emotional status
Integration group	A combination of both theories was used, i.e. five stages of Sarah Langwe’s empowerment model and four stages of self-efficacy theory. For empowerment, introducing facilities available in the environment, social or professional communication, introducing freelance jobs, introducing some service centers and the way of preparing suitable foods were considered. For self-efficacy, mastery experience, vicarious experience, verbal persuasion somatic, and emotional status were considered.	Integration of theoretical steps and model factors on self-efficacy and empowerment

In order to increase self-efficacy in the second group, four important factors considered in the formation of self-efficacy theory, including mastery experience, vicarious experience, verbal persuasion, and somatic and emotional status, were considered (Table 1). ^26^

In the third group (integration group), a combination of both theories was used, i.e. five stages of Sarah Langwe’s empowerment model and four stages of self-efficacy theory (Table 1).

Questionnaires were filled out three times: once in the pre-intervention stage, the second time in a month and the third time three months after completing the intervention. Women were allowed to complete the questionnaire by their own. For the illiterate ones, the questionnaires were completed by face to face interview by the first author.

The questionnaire was prepared in two parts. The first part was about demographic questions, and the second part was quality of life questionnaire as a dependent variable. Quality of life was assessed using the menopausal quality of life (MENQOL) questionnaire. The original version of the questionnaire was made by Hilditch et al. (1996), from the Women’s Health Society of Toronto, Canada. MENQOL is a 29-item questionnaire with vasomotor, physical, psychosocial, and sexual dimensions. In their study, face validity and content validity were verified. Intra-class correlation coefficients were 0.37, 0.81, 0.79, 0.70, and 0.55 for the vasomotor, physical, psychosocial, sexual domains, and quality of life questions, respectively. Cronbach’s Alpha scores for physical, vasomotor, psychosocial, and sexual domains were 0.87, 0.82, 0.81, and 0.89, respectively. The construct validity of the questionnaire was assessed using the discriminative validity method that was 0.69 for the physical, 0.66 and 0.40 for the vasomotor, 0.65 and -0.71 for the psychosocial, 0.48 and 0.38 for the sexual domains, and 0.57 for the quality of life questions. ^27^ Rostami et al. (2001) translated the questionnaire into Persian and modified it by panel of experts. Calculation of the correlation coefficient of scores of the two times completing the questionnaire in the test-retest did not show a significant difference before and after the test (r=0.92). Internal correlation about the questions showed a high correlation in three cases, that according to the common understanding of the study population of the questions, they were merged. ^28^ We did not find a study in Iran that evaluated the construct validity of this questionnaire. The Persian version of the questionnaire contains 26 questions. Questions were divided into four domains as: vasomotor (questions 1 and 2), psychosocial (questions 3-9), physical (questions 10-24), and sexual (questions 25-26) using four-point Likert scale. Zero was considered as none, 1 as minor, 2 as medium, and 3 as severe. The overall score was obtained by summing the scores of the questions, which was between zero to 78; the lower score indicated a better quality of life. This is a standard questionnaire and has been used in Kashan and Qasvin previously. ^15 , 29^ In the present study, quantitative content validity was assessed through content validity ratio (CVR) and content validity index (CVI) which were obtained over 0.70 and 0.70, respectively. Reliability was assessed using Cronbach’s Alpha that was 0.85. 

The obtained scores were described using mean (±standard deviation) for quantitative variables (age, the number of years since menopause, number of pregnancy and number of family members) and frequency (percentage) for qualitative variables (Education and Occupation). The homogeneity of the training groups before the intervention in terms of demographic variables was examined for quantitative variables using ANOVA test and for qualitative variables using Chi-square test. Repeated measure analysis was used to assess the impact of the intervention on the quality of life in three times completing the questionnaires, and Bonferroni post hoc test was used to compare the times. We used the Intention-To-Treat (ITT) analysis and reported the results only based on the perprotocol. All statistical calculations were performed using SPSS 22 software, and in all tests, a maximum error of 5% was accepted. 

Ethical considerations: The study was approved by the ethics committee with the code of IR.KAUMS.NUHEPM.REC.1398.061. Participation in the study was voluntarily. Also, women could withdraw from the study at any time. The questionnaires were anonymous, and women were told that their information would be kept confidential and used only in general. Written informed consent was obtained from the participants. For the illiterate participants, the questionnaire was completed by interview.

## RESULTS

Initially, 62 subjects entered each intervention group. During the study, a number of women did not complete the study due to the length of the intervention and the number of classes, lack of continuity, and incompleteness of the questionnaire (4 in empowerment, 3 in self-efficacy, and 6 in integration groups). Finally, 58 women in the empowerment group, 59 women in the self-efficacy group, and 56 women in the integrated group were included in the analysis.

Table 2 shows the demographic characteristics of postmenopausal women participating in the study. 

**Table 2:** Demographic characteristics of postmenopausal women participating in the study

Characteristic		Group			P
		Integration N=62	Self-efficacy N=62	Empowerment N=62	
		Mean±SD	Mean±SD	Mean±SD	
Age		53.08±3.36	54.20±3.93	53.37±4.08	*0.25
The number of years since menopause		3.33±2.10	3.97±3.22	4.05±3.21	*0.34
Number of pregnancy		3.68±1.68	4.19±3.19	3.80±1.68	*0.4
Number of family members		3.58±1.35	2.98±1.32	3.16±1.21	*0.03
		**N (%)**	**N (%)**	**N (%)**	
Education	Illiterate and elementary	51(82.25)	49(79.03)	58(93.55)	**0.04
	High school and above	11(17.75)	13(20.97)	4(6.45)	
Occupation	Housewife	54(87.09)	55(87.71)	57(91.93)	**0.46
	Worker	3(4.83)	5(8.06)	1(1.61)	
	Free and retired	3(4.83)	1(1.61)	1(1.61)	
	No answer	2(3.25)	1(2.52)	3(4.85)	

* ANOVA,

** Chi-square

The quality of life of all surveyed women in the first measurement was 32.97±11.61. The Kolmogorov-Smirnov test showed that the score of quality of life was normal in the first measurement, and in three groups (P=0.66 for empowerment, P=0.76 for self-efficacy, and P=0.77 for integration groups). According to Table 2, the three groups were similar in terms of the intervention factors, except “number of family members” and “level of education” that had a significant difference between groups; thus, these two variables were adjusted. Baseline data were entered as a confounder in repeated measure test. 

Given that Mauchly’s test of Sphericity was accepted at the 0.05 level (P=0.10), the Sphericity test was used to evaluate the subjects’ internal effects, which was significant during times (P<0.001). Therefore, the intervention has been effective in improving the quality of life of all three groups. In other words, improvement of the quality of life was statistically significant in three evaluations. 

Repeated measurements showed that the group effect was not significant, but the quality of life improved in the three intervention groups. Table 3 shows the comparison of mean±SD for quality of life at different times in the study groups (P=0.38 for Time*group, P=0.92 for group, and P<0.001 for Time). The results showed that the quality-of-life score decreased after the intervention, which indicates improvement in the quality of life. No different results were obtained from ITT test, and the interaction of group time was not significant; thus, the results were reported only based on the perprotocol.

**Table 3:** Comparison of mean±Standard deviation quality of life at different times in study groups

Groups	Evaluation time^a^			Repeated measure			Evaluation time comparison	*P value
	Tim 1 Mean±SD	Tim 2 Mean±SD	Tim 3 Mean±SD	Time	Group	Time× group		
Empowerment group	32.96±10.62	28.55±11.03	26.50±11.07	P <0.001	P=0.92	P=0.38	Tim1-Time2	<0.001
Self-efficacy group	31.93±12.54	29.06±11.72	28.03±12.27				Tim1-Time3	<0.001
Integrated group	34.07±11.70	28.71±12.79	27.94±11.51				Tim2-Time3	0.274

a Evaluation time: 1=pre-intervention, 2=one month after completing the intervention, 3=three month after completing the intervention,

* Bonferroni

## DISCUSSION

This study was designed to determine the effect of self-care education based on the self-efficacy theory, individual empowerment model, and their integration on the quality of life of postmenopausal women. The results of the study on each of the educational topics are discussed separately, including self-care training that was done similarly in three groups, empowerment in the first group, self-efficacy in the second group, and integration of empowerment and self-efficacy in the third group. 

After the trainings in all 3 groups, the quality of life improved and the decrease in the scores of the questionnaire used in the study confirms this. The MENQOL questionnaire was used for assessing the quality of life, which measures menopausal symptoms and shows a lower score for better quality of life. Other studies have previously shown that menopausal care training and life skills training improve the quality of life and self-care in postmenopausal women. ^10 , 25^ Moreover, self-care counseling for postmenopausal women improves their quality of life and lack of information is a major challenge in resolving menopausal problems. ^5 , 13 , 22^ The results of other studies are similar to ours because these studies, like ours, trained the participants about menopause-related self-care. The self-care training that all three groups received similarly in the first two sessions of the present study provided the important information they needed about menopausal self-care. 

In this study, empowerment training held for menopausal women in the empowerment group improved their quality of life. Similar to our results, empowering postmenopausal women improves their quality of life three months after the intervention in another study. ^15^ Also, it was previously shown that it is necessary to improve the menopause women’s quality of life and plan for their empowerment. ^14^ In addition, another study showed that middle-aged women should control chronic diseases such as high blood pressure, osteoarthritis, heart disease, and diabetes. ^13^ The similarity of these studies with the present one is that women gained the necessary ability for self-care by obtaining the necessary information, and this ability helped them in self-care and improved their quality of life. In order to empower women in the present study, like mentioned study, we trained the women about factors affecting health, such as obtaining health information, having proper nutrition, doing exercise, knowing how to access health centers, and having a job. They were also trained to control chronic diseases for empowerment. Since chronic diseases are screened and monitored in health centers in Iran, women were informed about this in the present study, and they were encouraged to use these services.

In the current study, self-efficacy training in the second group improved the women’s quality of life. This result is similar to other studies because it was shown that there was a positive correlation between quality of life and perceived self-efficacy. ^20 , 30^ All these studies confirm that women can overcome menopausal problems by improving the condition of four important factors that affect the formation of self-efficacy. These four factors include remembering the experience of previous successes, facing with other women who were successful in controlling menopausal problems, following verbal persuasion and accepting menopause, and accepting needs to control the menopause problems emotionally. ^26^ In the present study, all these steps and trainings to increase self-efficacy were considered, and self-efficacy training could improve the quality of life. 

Moreover, training of integration of women’s empowerment and self-efficacy for self-care in the third group improved the quality of life. It has already been shown that empowerment intervention has an effect on increasing self-efficacy. ^31^ In addition, in an interventional study in Mexico, a medical team of physicians, nurses, psychologists, and women themselves improved their quality of life through women’s empowerment for self-care. The self-efficacy of these women also improved following the improvement of their empowerment and self-care. ^11^ It can be said that empowerment by increasing self-efficacy can also affect the quality of life, and the present study has used an integration of these two trainings. It is better to say that women who have the necessary abilities are more confident, and they can take care of themselves. Also, integration of these two trainings can have a greater impact on women’s self-care and quality of life.

In the present study, the difference in quality of life after the intervention was not significant between the groups. In contrast in another study, the quality of life of postmenopausal women improved after training in the intervention group compared to the control group in vasomotor, psychosocial, sexual, and physical dimensions. However, there was a significant difference between the groups in their study instead of the result of present study. ^2^ It should be noted that in their study there were 5 training sessions instead of 4 sessions in the present study. They had an extra session and trained the women’s spouses and best friends, which added social support to effective education. In addition, we did not have a control group because the present study compares three training programs. Also, in the present study, there were only two dedicated sessions for each group, and half of the training sessions were about menopausal problems similarly between the three groups. Thus, if there were more dedicated sessions for each group or control group, perhaps a significant difference would be shown.

In the present study, assigning the participants to groups from different health centers reduced the likelihood of information transfer, and this was the strength of this study. Also, valid MENQOL questionnaire that was used in the study covered all domains of common symptoms of menopause. Participants were similar in terms of job status and most demographic characteristics. This makes the study population not scattered enough to make validation of the results better. The limitation of this study was that the sessions were held in health centers and in the morning, which made it impossible to involve employed women in the morning.

## CONCLUSION

The present study showed that trainings on empowerment in menopausal women as well as enhancing their self-efficacy for self-care can improve their quality of life. Introducing the services available in the community and teaching physical and mental change and self-care training can be used to increase the level of health of menopausal women. The results of this study can be used for health policy makers to plan health services and for health center staff to plan educational interventions. The results can also be used to prepare training packages to increase the empowerment and self-efficacy of postmenopausal women. Similar studies are recommended for menopausal women with chronic conditions such as diabetes and high blood pressure.

## ACKNOWLEDGEMENT

The study has been approved by Kashan University of Medical Sciences with the code of 98185. We would like to appreciate Kashan University of Medical Sciences for approving and financially supporting this project and also all women who participated in the study. 

**Conflict of Interest:** None declared
